# Prevalence of Dental Trauma and Their Relationship to Risk Factors among 8–15-Year-Old School Children

**DOI:** 10.1155/2022/3343827

**Published:** 2022-12-22

**Authors:** Kanamarlapudi Venkata Saikiran, Deepa Gurunathan, Sivakumar Nuvvula, Raghavendra Kumar Jadadoddi, Raichurkar Hemanth Kumar, Uday Chowdary Birapu

**Affiliations:** ^1^Department of Paediatric and Preventive Dentistry, Narayana Dental College and Hospital, Nellore, Andhra Pradesh, India; ^2^Department of Paediatric and Preventive Dentistry, Saveetha Institute of Medical and Technical Sciences, Saveetha Dental College and Hospitals, Saveetha University, Chennai 600077, Tamil Nadu, India; ^3^Department of Paediatric and Preventive Dentistry, SVS Institute of Dental Sciences, Mahabubnagar, Telangana, India

## Abstract

**Background:**

Tooth injuries lead to functional, aesthetic, and psychological disorders, accompanied by the great concern of the child, the parents, and the dentist.

**Aim:**

(a) To assess the prevalence of traumatic dental injury (TDI) and its relationship to risk variables among 8–15-year-old school children in Mahbubnagar, India. (b) To collect baseline data as there are limited reports of TDI studies in South India to date.

**Methods:**

A cross-sectional study was conducted among 6643 children from 78 schools in Mahbubnagar using a multilevel random sampling method. The permanent incisors were examined according to the WHO classification using a standard oral mirror and probe. Individuals with clinical evidence of trauma were asked about the details of the injury event using a structured questionnaire. The chi-square test analyzed the distribution of all measurements in this study with a statistical significance of 0.05.

**Results:**

Among the 6643 children from the 78 schools surveyed, 9.3% experienced TDI. TDI occurred in 68% of boys, which was about twice as high in girls at 32%. The most commonly affected teeth were the maxillary central incisors. A higher number of children with an incisal overjet more significant than 3 mm had TDI than the children less than 3 mm, although this difference was not statistically significant. The lip closure incompetence was more common in children with TDI. The most frequent causes of TDI were falls, and the site of occurrence was school. Type I fractures were the most prevalent and most went untreated.

**Conclusion:**

The high level of dental trauma and the low percentage of children with trauma seeking treatment emphasize the need for greater awareness among the Mahbubnagar children.

## 1. Introduction

Dental trauma is the most common health problem with a worldwide. According to a global systematic analysis by Petti et al. there are roughly 900 million people worldwide between the ages 7 and 65 who have damaged permanent teeth. 180 million children around the world were expected to have at least one primary tooth-related TDI [1]. Nearly 80% of dental injuries occur under the age of 20, with two peak incidences in boys, ages 1–3 years and 10–12 years, and a peak in girls at age 13, making childhood and adolescence very vulnerable to traumatic dental injuries [2]. An untreated and unsightly fracture of an anterior tooth can affect a child's behaviour and progress at school and in daily life [3]. The literature suggests that children who have experienced trauma to their anterior teeth are more prone to avoid smiling and laughing and being more self-conscious than children without dental trauma [4]. Similarly, children with untreated dental trauma experience social isolation and embarrassment and are recognized to have a deprived oral health-related quality of life [5]. It has been documented that adolescents with broken teeth are 20 times more disruptive in everyday life than adolescents without dental trauma [6]. Parents often ignore these injuries to primary dentition concerning dental crowns. Still, in severe form, the damage to supporting structures such as the alveolar bone is the cause of the first visit to the emergency services. The majority of dental injuries affect the front teeth [7, 8]. The upper incisors are the most commonly affected teeth because they are exposed in the dental arch. Children who are male, have prognathic maxillae, or have a noticeable overjet are more likely to sustain cutting injuries [9]. The aetiology of dental trauma is significantly influenced by several variables, including age, socioeconomic level, and environmental effects [10].

The prognosis of teeth after the injury depends on the type of TDI, the emergency treatment, and the time elapsed until the final restoration [11]. Low awareness among the general public and physicians usually leads to delays in finding treatment possibilities, pain, severe symptoms, and poor prognosis. Traumatic injuries to permanent teeth have been reported to have a prevalence rate between 6.1% and 58.6%. The wide variation in reported rates can be attributed to several factors, including study type, trauma classification methodology, study size and population, geographic location, and differences in cultural behaviour [12]. Many studies on the prevalence of traumatic dental injuries are reported in the literature in India [13–17]. Traumatic dental injuries are a severe public dental health problem among children in underprivileged areas. To obtain a more comprehensive view of oral health, it is crucial to gather data locally on dental injuries [3]. No literature on the aetiology and prevalence of traumatic injuries to anterior teeth in Mahbubnagar children is available. Hence, the present study was planned to determine the prevalence of dental trauma in anterior teeth and their relationship to predisposing risk factors in 8–15-year-old children. The prevention and policy strategies are also proposed.

## 2. Materials and Methods

### 2.1. Ethical Approval

Institutional ethical clearance for the study was obtained, formal letters were sent to the selected schools, and the approval of the school authorities was obtained. School directors and teachers have been informed of the curriculum for the support and collaboration. All parents or guardians were asked to sign a written informed consent form outlining the study's aims, characteristics, and significance.

### 2.2. Study Design and Population

A cross-sectional study was conducted using a questionnaire and clinical examination of the upper and lower permanent incisors (eight teeth) in 8–15-year-old children who regularly attend government and private schools in urban and rural areas of Mahbubnagar, and the study period was from January 2019 to January 2020.

### 2.3. Data Collection

The map of Mahbubnagar city was adopted and divided into five zones, namely, east, west, north, south, and central zones, and schools were selected based on their location. In the first phase, a list of schools with addresses, telephone numbers, number of classes, and the total number of children was made. In the second stage, the schools were divided by area, i.e., private and public schools; in the third phase, an equal number of schools were randomly selected. Since the number of students differed from school to school, sampling with a probability proportionate to school size was used. The study included children whose permanent anterior teeth had erupted, or at least 3/4 of the crown had penetrated the oral cavity, and in the 8–12-year age group. Children with a history of dental trauma who had not sustained an injury more than once were included in the study. Students who had lost an anterior tooth due to dental caries, broken roots, severe dental fluorosis, and children with physical, mental, or medical disabilities were excluded from the study. Children who received or had undergone orthodontic treatment were excluded from the study.

### 2.4. Diagnostic Criteria of Traumatic Dental Injury

The children were seated in a chair, and the ADA type III assessment was performed using a disposable oral mirror and a Williams probe under adequate lighting. Strict infection control measures have been applied. Four trained paediatric dentists collected all information on standardized trauma assessment forms according to the diagnostic criteria. Additional details such as the cause and place of injury were noted for the children who underwent dental trauma. The teeth were examined by the direct vision. Neither vitality nor radiographs were used to assess the extent of the fractured teeth. The examination was uniformly performed from the maxillary right quadrant to the mandible in a clockwise direction using the Ellis and Davey trauma classification [18].

The overjet was measured with a Community Periodontal Index probe, which was dichotomized between 3 mm and >3 mm during the analysis. The overjet was calculated from the lingual incisal line angle of the most prominent upper incisor to the buccal aspect of the corresponding lower incisors. The CPI probe was used to measure the degree of overjet as described in 1997 WHO Basic Oral Health Survey Guidelines. The lip covering was measured using the standards established by burden. If the lip covered the upper incisors at rest, the lip coverage was rated as sufficient. The lip coverage was rated insufficient if most upper incisors were exposed or the lip strain was evident upon closure.

All the obtained data were entered into a Microsoft Excel spreadsheet and subjected to statistical analysis at the end of the study. The data were analyzed with SPSS version 20. The descriptive analysis was performed for frequency distribution. Means and standard deviations of continuous variables such as age were calculated. A chi-square test was used for bivariate analysis, and multiple logistic regression was performed to assess the association between dental injury occurrence and gender, incisal overjet, and lip coverage. The level of significance was *p* < 0.05.

## 3. Results

### 3.1. Prevalence

A total of 6643 children were examined. The response rate was 100%, and the sample size satisfied the requirements. The group consisted of 3774 boys (56.8%) and 2869 (43.2%) girls. 737 were eight years old, 834 were nine years old, 895 were ten years old, 871 were 11 years old, 860 were 12 years old, 835 were 13 years old, 795 were 14 years old, and 816 were 15 years old.  Table 1 summarizes the sample distribution of TDI according to age and gender.

The prevalence of permanent anterior teeth TDI was 9.3%, affecting 645 school children of both genders. 439 (68%) were boys and 206 (32%) were girls. A statistically significant difference between genders was found boys were 2.1 times more prone to traumatic dental injuries than girls (*p* < 0.05), as illustrated in Table 2. 421 (65.27%) had one injured tooth, while 224 (34.73%) had two injured teeth. Maxillary central incisors (74.58%) were the most commonly affected fractured teeth, followed by maxillary lateral incisors (25.42%). Out of the two injured teeth, both maxillary central incisors were involved in 82.47% of children; maxillary central and lateral incisors were involved in 17.53%.

Table 3 summarizes the frequency distribution of types of TDI in school children. The most common type of fracture was a simple fracture of the crown involving little or no dentin (class I), which accounted for 45.89%, and the least common was missing teeth due to trauma (4.34%), followed by others. This association was determined to be statistically significant (*p* = 0.05).

### 3.2. Causes and Place of TDI

The fall was the most common cause of injury (51.2%) for most children, and biting (4.8%) was the least common cause of trauma to the teeth in children, as illustrated in Table 4. The majority of the injuries occurred at school (50.69%), followed by home (42.32%) and other places (6.99%), such as streets and playgrounds, as shown in Table 5. With a *p* value of 0.001, the association of the place with the occurrence of traumatic injury was significant.

### 3.3. Risk Factors

Table 6 displays the logistic regression findings, which revealed a strong correlation between gender, the amount of overjet, the type of lip coverage, and dental trauma. 5.23 per cent of the patients had TDI when their overjet was 3 mm or higher compared to 1.5 per cent of the subjects when their overjet was less than 3 mm (*p*= 0.05). The multiple logistic regression analysis revealed that children with an overjet size >3 mm (95 per cent CI = 0.624–0.898) were more likely to present with a tooth injury. Increased overjet had an odds ratio of 0.684. In comparison to the subjects with competent lip closure, subjects with incompetent lip closure showed a substantially higher number of TDI (*p* 0.05).

### 3.4. Treatment Modality

Only 11.89% of the affected children had sought treatment for their dental injuries, compared to 64.85% who did not receive any treatment as they had no problem with TDI. Another important finding was that 23.25% did not receive any treatment as they were unaware of the injury. The other reasons for not receiving the treatment were disinterested parents (12.25%), high cost (6.12%), and fear of pain (4.90%).

## 4. Discussion

The prevalence of traumatic dental injuries has been found to vary considerably, ranging from 4% to 58% in several epidemiological studies [19]. The prevalence of TDI with permanent anterior in the present study was found as 9.3% by examining almost six thousand children aged 8–15. The prevalence revealed in this study was lower than the prevalence reported by authors from different parts of India, such as Gupta et al. (13.8%), Dua and Sharma (14.5%), Baldava and Anup (14.9%), and Ravishankar et al. (15.1%) [16, 20–22]. When examining children of a similar age, Patel and Sujan (8.79%) and Ain et al. (9.3%) reported the prevalence same as the present study [14, 23]. Nik-Hussein and Gupta et al. reported a low prevalence between 4.1% and 4.15%, respectively [24, 25]. These discrepancies in the reported prevalence from various studies can be attributed to factors such as the study's design, sample size and sampling procedure, diagnostic criteria, restricted age groups, and behavioural and geographical differences between the study locations [26].

In the present study, the prevalence of TDI was higher in boys than in girls, which is consistent with other studies where male individuals have a higher chance of TDI than female individuals [14, 27, 28]. This increased the prevalence in boys that can be attributed to their active participation in physical activities that require more strength, such as contact sports, fights, rough play without adequate protection, and apparatus with higher risk potential. The discrepancy between boys and girls might be due to the constrained behaviour of girls imposed by conservative parents owing to the cultural and social circumstances in India. Girls are more mature in nature than boys at an earlier age because of adjourned pubertal development in boys, which could also be a contributing factor [14, 16]. In comparison, some studies exhibited no significant differences, while some studies quantified an increasing tendency of TDI in girls, which can be attributed to the increased engrossment of girls in sports and physical relaxation activities that were formerly constrained only to boys [15, 29]. In addition, girls can also be exposed to violence and road traffic accidents similarly to boys [30].

Out of all 645 children presented with the TDI in the present study, most of the children exhibited injury to one tooth (296) and two teeth (218), per previously stated studies [31, 32]. The aetiology of the injury may differ according to the child's age, sex, and socioeconomic status. Similarly, in the present study, “falls” (51.2%) tailed by “sports” (20.4%) were the most common etiological factors associated with dental trauma, which is analogous to other stated studies [33–37]. Most traumatic dental injuries were acquired at home and tracked by the school. Other studies attained similar results. The most common events commenced by the children at the time of the accident were leisure and sports activities. Most injuries to children (especially girls) might be because they spend more time at home than school [28]. The importance of the school environment must be accentuated as children were more indulged in physical and social activities, which was also strongly associated with dental trauma after the home environment [38].

In larger cities, schools with a more significant number of children in each class, and in total, specific measures for the prevention or reduction of the incidence of TDIs were maintained, conversely for schools in rural areas, where levels of commitment towards health along with the safety were low [39]. The association between the overjet and TDI has been sightseen largely by various authors, which yielded differing results. In the present study, children with overjet more than 5 mm revealed a significant association and had a greater risk of TDIs than those with overjet less than 5 mm. Arraj et al. in a systematic review and meta-analysis stated that children in their early secondary dentition with an overjet ≥5 mm could be regarded as the threshold for trauma [40].

Schatz et al. stated that the overjet was the most significant factor with an augmented risk of the tooth trauma after traumatic injury. An increased overjet of 6 mm or more significantly impacted the risk of trauma [41]. Petti et al. stated that individuals could have a 2.5-fold higher risk for TDI if they have an overjet exceeding 3 mm than those with the standard [42]. In the present study, children with inadequate lip coverage had five times more TDIs than those with the adequate lip coverage. The protective effect of the lip and good occlusion of maxillary and mandibular teeth tends to diminish the impacting force of trauma [43]. Thus, the inadequate lip coverage has been revealed as an essential risk factor and a significant determinant for TDIs in this study [9, 22]. Patel and Sujan, in their cross-sectional survey stated that children with the adequate lip coverage showed less number of injuries (5.32%) compared to those with inadequate lip coverage (24.19%) and stated that children with insufficient lip coverage were 5.4 times more prone to TDI [14].

## 5. Conclusion

The prevalence of TDI in this population was found to be 9.30%. Class I fracture was the most common type of injury (45.89%), and most were untreated. The school-going children in Mahbubnagar city need to receive dental education to raise awareness of the dental health, thereby improving the quality of life in children. Proper educational programs should be planned to educate parents regarding the prevention and importance of treating early traumatic dental injuries for a better outcome.

## Figures and Tables

**Table 1:** The prevalence of the traumatic injuries according to the age and gender.

Age	Male		Female		Total	
	TDI absent	TDI present	TDI absent	TDI present	TDI absent	TDI present
8 years	382	39	296	20	678	59
9 years	409	55	342	28	751	83
10 years	432	69	362	32	794	101
11 years	441	62	344	24	785	86
12 years	428	58	348	26	776	84
13 years	434	53	319	29	753	82
14 years	396	51	324	24	720	75
15 years	413	52	328	23	741	75

**Table 2:** Boys:girls ratio and the prevalence of boys with anterior teeth fracture.

Gender	Number of children examined	Number of children with TDI	Gender ratio	Prevalence (%)	*P* value
Boys	3774 (56.8%)	439 (68%)	2.1 : 1	9.30	0.001^*∗*^
Girls	2869 (43.2%)	206 (32%)			
Total	6643	645			

^*∗*^: significant.

**Table 3:** The frequency distribution of types of traumatic dental injuries in school children.

Status of trauma	Number of injured teeth (%)	Boys with injured teeth	Girls with injured teeth	*P* value
1	296 (45.89%)	189 (43.05%)	107 (51.94%)	0.001^*∗*^
2	218 (33.79%)	154 (35.07%)	64 (31.06%)	
3	64 (9.92%)	46 (10.47%)	18 (8.73%)	
4	39 (6.04%)	29 (6.61%)	10 (4.86%)	
5	28 (4.34%)	21 (4.78%)	7 (3.39%)	
Total	645	439	206	

^*∗*^: significant.

**Table 4:** The distribution and percentage of children with traumatized anterior teeth according to the place of occurrence of trauma.

Etiology	Children with injured teeth (%)	Boys with injured teeth	Girls with injured teeth	*P* value
Fall	334 (51.2%)	217 (49.43%)	117 (56.79%)	0.001^*∗*^
Sports	132 (20.4%)	94 (21.41%)	38 (18.44%)	
Road traffic accident	85 (13.1%)	62 (14.12%)	23 (11.16%)	
Collision	63 (9.8%)	46 (10.47%)	17 (8.25%)	
Biting	31 (4.8%)	20 (4.55%)	11 (5.34%)	
Total	645	439	206	

^*∗*^: significant.

**Table 5:** The distribution and percentage of children with traumatized anterior teeth according to the place of injury.

Place	Children with injured teeth	Boys with injured teeth	Girls with injured teeth	*P* value
Home	273 (42.32%)	137 (31.20%)	136 (66.01%)	0.001^*∗*^
School	327 (50.69%)	268 (61.04%)	59 (28.64%)	
Others	45 (6.97%)	34 (7.74%)	11 (5.33%)	

^*∗*^: significant.

**Table 6:** Multiple logistic regression analyses of gender, size of the incisal overjet, and type of lip coverage for TDI.

Parameters		Traumatic dental injury		*P* value	OR and CI
		Yes (%)	No (%)		
Gender	Boys	439 (7.31%)	3335 (55.60%)	0.001^*∗*^	(1.615), 1.407–2.149
	Girls	206 (3.43%)	2663 (44.40%)		

Overjet	Less than 3 mm	94 (1.5%)	5904 (98.43%)	0.001^*∗*^	(0.684), 0.624–0.898
	More than 3 mm	314 (5.23%)	5684 (94.77%)		

Lip coverage	Adequate	49 (0.81%)	5935 (99.19%)	0.001^*∗*^	(1.473), 1.034–2.008
	Inadequate	359 (5.98%)	5639 (94.02%)		

^*∗*^: significant.

## Data Availability

The analyzed data during the current study are available from the corresponding author on reasonable request.
